# Inactivated FABP5 suppresses malignant progression of prostate cancer cells by inhibiting the activation of nuclear fatty acid receptor PPARγ

**DOI:** 10.18632/genesandcancer.192

**Published:** 2019-05

**Authors:** Waseem Al-Jameel, Xiaojun Gou, Xi Jin, Jiacheng Zhang, Qiang Wei, Jianzhong Ai, Hong Li, Asmaa Al-Bayati, Angela Platt-Higgins, Andrew Pettitt, Philip S. Rudland, Youqiang Ke

**Affiliations:** ^1^ Department of Molecular and Clinical Cancer Medicine, Liverpool University, Liverpool, United Kingdom; ^2^ Sichuan Antibiotics Industrial Institute, Chengdu University, Chengdu, China; ^3^ Institute of Urological Research, West China Hospital, Sichuan University, Chengdu, China; ^4^ Department of Biochemistry, Liverpool University, Liverpool, United Kingdom; ^5^ Department of Pathology, College of Veterinary Medicine, University of Mosul, Mosul, Iraq

**Keywords:** FABP5, dmrFABP5, PPARγ, CRPC, tumorigenicity

## Abstract

Previous study has suggested that the FABP5-PPARγ-signalling transduction pathway gradually replaces the androgen receptor activated pathway in promoting malignant progression of castration-resistant prostate cancer (CRPC) cells. To interfere with this newly discovered FABP5-related signalling pathway, we have produced a highly efficient recombinant FABP5 inhibitor, named dmrFABP5. Treatment with dmrFABP5 significantly supressed the proliferation, migration, invasion and colony formation of the highly malignant prostate cancer cells PC3-M *in vitro*. To test dmrFABP5's suppressive effect in CRPC, the human PC3-M cells were implanted orthotopically into the prostate gland of immunosuppressed mice to produce tumours. These mice were then treated with dmrFABP5 and produced a highly significant reduction of 100% in metastatic rate and a highly significant reduction of 13-fold in the average size of primary tumours. Immunocytochemial staining showed that the staining intensity of dmrFABP5 treated tumours was reduced by 67%. When tested *in vitro*, dmrFABP5 suppressed the cancer cells by blocking fatty acid stimulation of PPARγ, and thereby prevented it activating down-stream cancer-promoting or inhibiting cancer-suppressing genes. Our results show that the FABP5 inhibitor dmrFABP5 is a novel molecule for treatment of experimental CRPC and its inhibitory effect is much greater than that produced by SB-FI-26 reported in our previous work.

## INTRODUCTION

Prostate cancer is common in countries with a high dietary consumption of fatty acids [1]. In the early stage, growth and dissemination of prostate cancer cells depend on androgen supplied through the peripheral blood and thus therapy has been directed towards deprivation of androgen. Androgen-deprivation therapy (ADT) is initially very effective. However, in most cases the disease relapses within 2–3 years with recurrence of lethal castration-resistant prostate cancer (CRPC). This disease cannot be treated effectively by ADT [2]. The CRPC cells overexpress fatty acid synthase (FASN) and acetyl-CoA carboxylase, which are key enzymes involved in the synthesis of fatty acids [3–5]. Fatty acids are not only active components of many biological processes, but also are essential signal transduction molecules in pathways involved in advanced prostate cancer progression [6–8]. Thus blocking the supply of fatty acids has become a therapeutic strategy to suppress malignant dissemination of prostate and some other cancer cells [9].

Fatty acid-binding protein 5, or FABP5, is a 15kDa cytosolic protein, which binds with a high affinity to medium and long chain fatty acids [10]. After FABP5 had been shown to promote malignant progression in prostate cancer cells [11–14], its increased expression in archival prostate cancer tissues was found to be significantly associated with reduced patient survival times. Thus FABP5 is now a valuable prognostic factor for advanced prostate cancer [15]. Moreover, recent investigations have established that there is a novel fatty acid-initiated signalling pathway that leads to malignant progression of prostatic cancer cells. Thus, when FABP5 expression is increased, excessive amounts of fatty acids are transported into the nucleus of the prostate cancer cells, where they act as signalling molecules to stimulate their nuclear receptor PPARγ. The activated PPARγ then modulates the expression of its down-stream regulatory genes and these changes finally lead to enhanced tumour expansion and aggressiveness caused by an overgrowth of cells with increased angiogenesis and reduced apoptosis [16].

If the malignant progression of CRPC cells can be suppressed by inhibiting the biological activity of FABP5, the availability of highly effective inhibitors to FABP5 is an important first step in testing this hypothesis. Inhibition of FABP5's activity has been shown by using chemically synthesized inhibitors, e.g. BMS309403, to be effective for treatment of inflammatory and metabolic diseases [17–20]. Recently-developed FABP5 inhibitors, which produce approximately 50% of the inhibitory effect of BMS309403, were originally effective analgesics and anti-inflammatory agents in mice [21–23]. These small molecule inhibitors included SB-FI-26 (α-truxillic acid 1-naphthyl mono-ester). This compound was tested in our previous work and exhibited a significant suppression of both primary tumour growth and metastasis of the CRPC cells [24]. SB-FI-26 is, in fact, the active component of a traditional herbal medicine (*Incarvillea sinensis*) which has been used to treat pain and rheumatism for hundreds of years [25, 26].

FABP5 binds to fatty acids through a binding motif that consists of 3 key amino acids [27]. Our previous work showed that the structural integrity of this fatty acid-binding motif is essential for the tumour-promoting function of FABP5 [16]. The mutant protein dmrFABP5 is a recombinant molecule generated by changing 2 of the 3 amino acids in the fatty acid-binding motif of FABP5. Thus dmrFABP5 has lost most of its ability of binding to and transporting fatty acids. In this work, we have tested the potential of dmrFABP5 as a bio-inhibitor of FABP5 for the treatment of CRPC in mice. Moreover, its suppressive effects have been compared to those of SB-FI-26 under the same conditions.

## RESULTS

### Production of recombinant FABP5s and testing their biological activities

**Figure 1:**
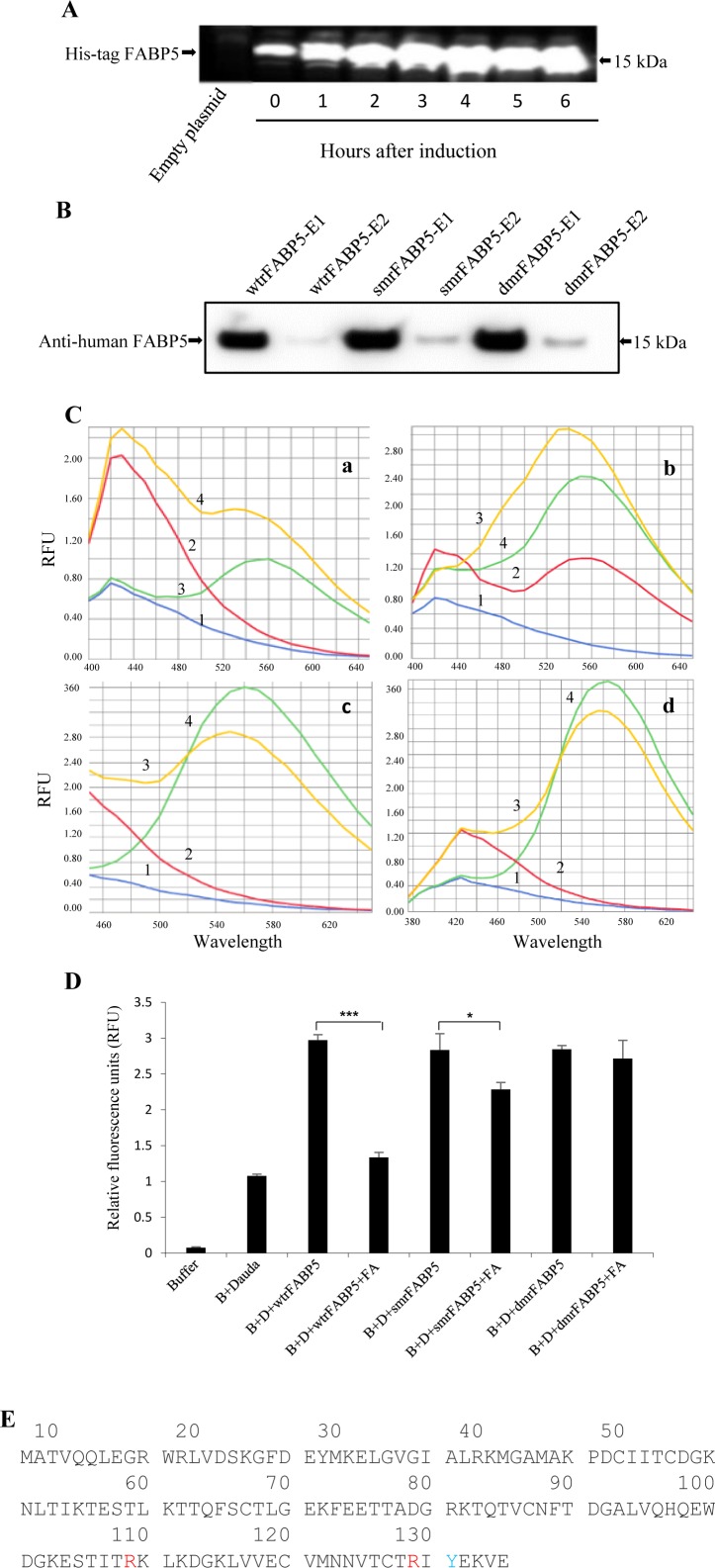
Production of recombinant FABP5s in E. coli cells and testing their binding affinity to fatty acids **A.** Determination by Western blot of the optimal time point at which the maximum amount of recombinant protein was synthesized in bacterial cells. 6×His-tag bound protein bands were recognized by the Penta-His antibody. The wtrFABP5 protein synthesized at different times is shown in 7 separate lanes. Bacterial cells harboring empty plasmid were used as a negative control. **B.** Western blot analysis of different recombinant FABP5s purified by affinity chromatography. Bands of FABP5 protein (collected in eluates E1 and E2) were identified by monoclonal anti-human FABP5. **C.** Representative graphic records of fatty acid binding properties of the recombinant FABP5s by DAUDA displacement assay. a) Effect of wtrFABP5 on the fluorescent emission spectra of a fluorescent fatty acid (DAUDA) ligand at the excitation wavelength of 345nm. Reaction solutions contained: (1) PBS, (2) wtrFABP5 in PBS, (3) 2μM DAUDA, (4) 2μM DAUDA with 3μM wtrFABP5. b) Competitive inhibition of DAUDA binding to wtrFABP5 with palmitic acid. c) Competitive inhibition of DAUDA binding to smrFABP5 with palmitic acid. d) Competitive inhibition of DAUDA-dmrFABP5 binding to palmitic acid. For a, b, and c: reaction solutions contained: (1) PBS, (2) 3μM X in PBS, (3) 3μM X and 2μM DAUDA, (4) 3μM X and 2μM DAUDA plus 2μM palmitic acid. X = wrtFABP5, smrFABP5 or dmrFABP5. **D.** Fluorescent intensities of displaced DAUDA from different recombinant FABP5s by palmitic acid as an indication of their relative fatty acid-binding ability. The value produced by the buffer and DAUDA plus FABP5s was set at 1 as control. The results (mean ± SE) were obtained from 3 separate experiments (2-tailed unpaired Student's *t* test, ^***^, *p* < 0.0001; ^*^p <0.05). **E.** Protein sequence of human FABP5 (Source: UniProtKB – Q01469, FABP5_HUMAN): Three key amino acids of the fatty acid-binding motif were highlighted.

All 3 constructs containing cDNAs for wtrFABP5, dmrFABP5 and smrFABP5, respectively, were transformed into competent BL21 *E. coli* cells for production of protein. After induction, the wtrFABP5 recognized by the anti-His tag antibody in the recombinant bacteria was gradually expressed and reached its maximum after 4 hours (Figure 1A). When extracts were Western blotted with a monoclonal anti-human FABP5, only a single band in the first fraction was detected for each of the 3 recombinant FABP5s indicating that each recombinant protein was of high purity. Small amounts of the recombinant protein were also present in the second elution fraction (Figure 1B). The fatty acid-binding affinity of wtrFABP5 to the fluorescently labelled fatty acid DAUDA was tested and its maximum affinity was detected at 530nm, this corresponded to a shift in DAUDA's original emission wavelength (Figure 1C/a). The fluorescent intensity decrease when DAUDA was displaced from each recombinant FABP5 by palmitic acid was used as an indication of their relative binding affinity. Addition of palmitic acid to the wtrFABP5-DAUDA complex created a noticeable drop in the fluorescent intensity (Figure 1C/b). However, addition of palmitic acid to smrFABP5- DAUDA complexes was able to produce only a small reduction in fluorescent intensity. Addition of palmitic acid to dmrFABP5-DAUDA complexes produced almost no reduction in fluorescent intensity (Figure 1C/c, d). When the level of fluorescent intensity of DAUDA (D) + Buffer (B) was set at 1 (Figure 1D), the fluorescent intensity of the complex of wtrFABP5, D and B without palmitic acid was 2.97 ± 0.08. When palmitic acid was added to the complex, the level of fluorescent intensity was significantly reduced by 83% to 1.34 ± 0.7 (Student's *t* test, *p* < 0.0001). Thus wtrFABP5 exhibited a strong ability to bind to palmitic acid and displaced 83% DAUDA. When palmitic acid was added to complexes of smrFABP5 + B +D, the level of fluorescent intensity was reduced moderately, but significantly by 30% (Student's *t* test, *p* < 0.01). However, when palmitic acid was added to complexes of dmrFABP5 + B +D, the level of fluorescent intensity was only slightly reduced by 7%, indicating that dmrFABP5 was able to replace only 7% of the DAUDA, thus dmrFABP5 had lost most of its ability of binding to fatty acids. Therefore, when Arg^109^ was changed to Ala^109^ (smrFABP5) and that change was combined with the change of Arg^129^ to Ala^129^ (dmrFABP5) (Figure 1E), these substitutions either partially or almost completely inhibited FABP5's ability of binding to fatty acids.

### Inhibitory effect of dmrFABP5 on malignant characteristics of PC3-M cells

**Figure 2:**
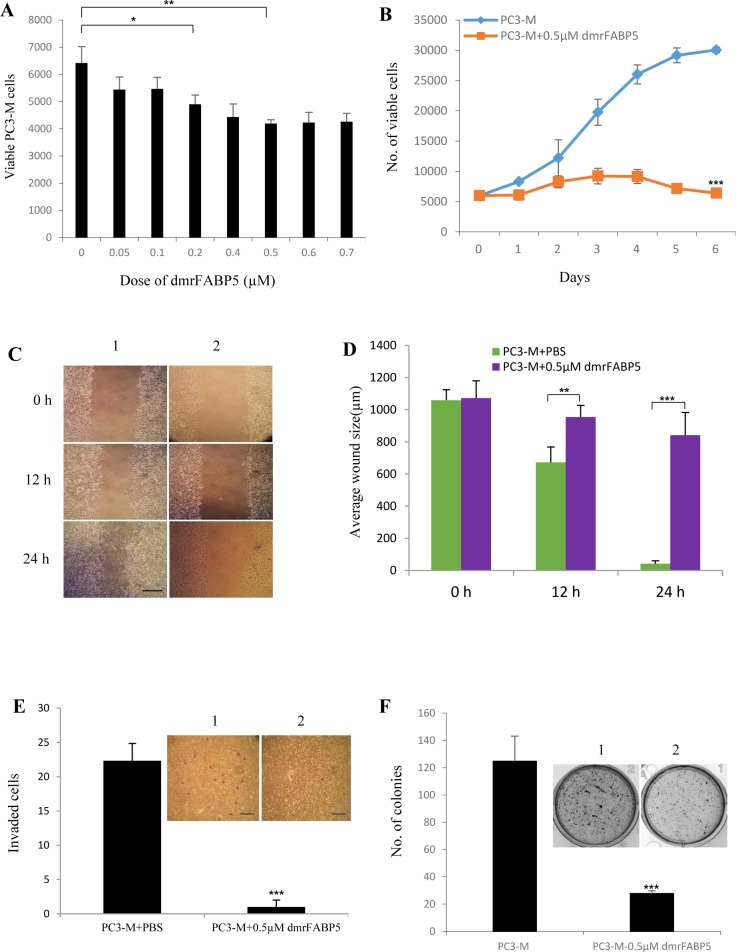
DmrFABP5 significantly suppressed the proliferation, migration, invasion and anchorage-independent growth of PC3-M cells **A.** Testing the optimal inhibitory concentration of dmrFABP5 by calculating viable cell numbers measured by the MTT assay. **B.** Inhibitory effect of 0.5μM dmrFABP5 on proliferation of PC3-M cells over the 6-day experimental period. **C.** Representative images of the wound healing assay. Cell migration capacity was measured by the reduction in wound size in control (1) and in cultures treated with 0.5μM dmrFABP5 (2) measured at 0, 12 and 24 hours after treatment. Scale bar is 250μm. **D.** Average wound sizes (μm) of the control PC3-M cells and of cells treated with 0.5μM dmrFABP5 at 0, 12 and 24 hours after treatment. **E.** Numbers of invading cells from the control (1) and from cells treated with 0.5μM dmrFABP5 (2) for 24h after different treatments. Results (mean ± SE) were obtained from three separate measurements. Scale bar is 250 μm. **F.** Colonies produced in soft agar by the control (1) and by cells treated with 0.5μM dmrFABP5 (2) 2 weeks after the different treatments. Results (mean ±SE) were obtained from three separate wells in each treatment. All *in vitro* results were subjected to 2-tailed unpaired Student's *t* test and ^*^, *p* < 0.05; ^**^, *p* < 0.001; ^***^, *p* < 0.0001.

The effects of dmrFABP5 on the malignant characteristics of the PC3-M cells are shown in Figure 2. Cytotoxicity tests showed that treatment of PC3-M cells with dmrFABP5 significantly suppressed their viability in a concentration-dependent manner. Maximum suppression was observed at 0.5μM dmrFABP5; further increases in concentration did not produce any further significant suppression. When treated with this optimal concentration, cell numbers were significantly reduced by 35% (Student's *t* test, *P* < 0.001) (Figure 2A). When the same cells were tested using a MTT assay, 0.5μM dmrFABP5 significantly reduced their proliferation rate by 4.7- fold (Student's *t* test, *P* < 0.0001) (Figure 2B). When tested in a cell migration assay (Figure 2C), the same cells treated with dmrFABP5 produced only a 21% reduction in wound size in 24h. These treatments significantly inhibited the migration rates of PC3-M cells (Student's *t* test, *p* < 0.0001), leading to only small wound closures for treated groups compared to an almost complete wound closure (94%) for the control (Figure 2D). When tested in an invasion assay, the mean number of invaded cells from the control PC3-M cells and the same cells treated with dmrFABP5 was 2 ± 1, representing a significant inhibition of invasion by 91% (Student's t test, *P* < 0.0001) (Figure 2E). Further tests in soft agar showed that the number of colonies formed after 2 weeks by control PC3-M cells and PC3-M cells treated with dmrFABP5 were 124 ± 18, and 23 ± 2, respectively, representing a highly significant inhibition by 81% (Student's *t* test, *p* < 0.001) (Figure 2F).

### Effect of dmrFABP5 on tumorigenicity and metastatic ability of PC3-M cells in mouse prostate gland

**Figure 3:**
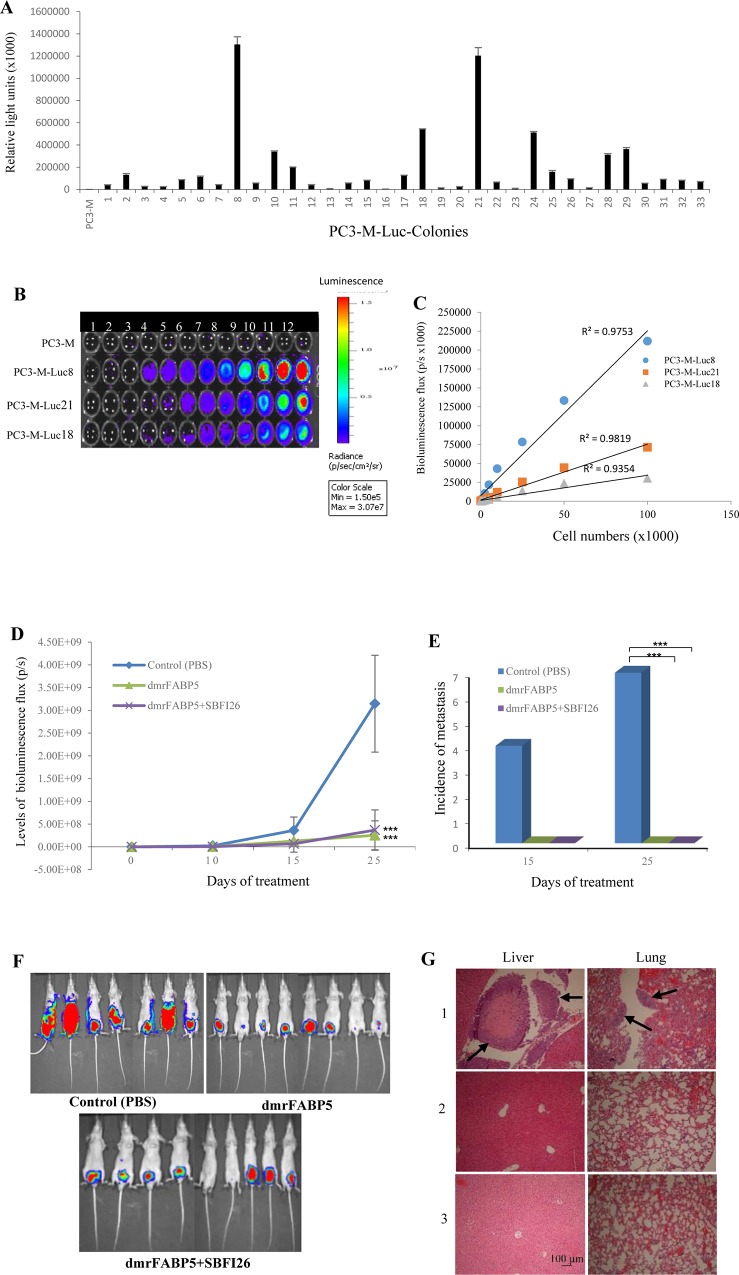
DmrFABP5 significantly inhibited the tumorigenic and metastatic ability of PC3-M cells implanted orthotopically into the prostate gland of nude mice **A.** Establishment of stable PC3-M colonies expressing strong bioluminescent signals. The three colonies which expressed the highest signals were identified as PC3-M-Luc8, PC3-M-Luc21 and PC3-M-Luc18. **B.** Relationship of the luminescent intensity to the number of cells was assessed by an IVIS imaging system. **C.** Correlation between the bioluminescent flux intensity (photons/second) and the number of cells derived from three different PC3-M-Luc colonies. **D.** Whole body tumour bioluminescent flux produced by each group of nude mice treated with: PBS (control), dmrFABP5 (20mg/kg), or a combination of dmrFABP5 (20mg/kg) and SBFI26 (1mg/kg) for 25 days. Although eight mice were used in each group, in one control mouse, the tumour mass grew too fast, so it reached 20% of its body size and caused pain to the animal at day 15. Thus this mouse was euthanized and autopsied prematurely, and hence was excluded from the resulting assessment. Values were plotted as mean ± SE (*n* = 7, 8, and 8); the differences were assessed by two-tailed unpaired Student's *t* test ^***^, *P* < 0.0001. **E.** Numbers of mice which developed one or more metastases in the control and the experimental groups after 15 or 25 days of treatment. The differences were assessed by 2-tailed Fisher's Exact test ^*^, *P* < 0.05. **F.** Ventral bioluminescence images of primary tumours and metastases in all three groups of mice 25 days of treatment. **G.** Liver and lung metastases (arrows) from mice which received injection of PBS (1), dmrFABP5 (2) or combination of SB-FI-26 and dmrFABP5 (3). Sections were H&E stained. Magnification ×10 and scale bar is 100 μm.

PC3-M cells were transfected with the luciferase vector and 3 colonies which generated high bioluminescent signals were picked and named PC3-M-Luc8, PC3-M-Luc21, and PC3-M-Luc18 (Figure 3A). PC3-M-Luc8 produced the highest level of bioluminescent signal (Figure 3B) and there was a significant correlation between total flux and the number of cells (R^2^ = 0.98) (Figure 3C). Luciferase-labelled PC3-M-Luc8 cells were implanted orthotopically into the prostate gland of each of 3 groups of male nude mice. The mice were then injected intraperitoneally daily with PBS, dmrFABP5, and a combination of dmrFABP5 and SB-FI-26, for 25 days. On day 25, there was a large decrease in bioluminescent signal in the dmrFABP5 group (2.53×10^8^ units of fleux-UF) and dmrFABP5 plus SB-FI-26 (3.67×10^8^ UF) group, compared with the control (31.5×108 UF). On the basis of bioluminescence, our results showed about a 13-fold and 9-fold suppression in tumor mass by the dmrFABP5 group and by the combined inhibitors group over that of the control group (Student's t test, *P* < 0.0001) (Figure 3D). In the control group, 7/7 (100%) mice produced metastases. In groups treated with dmrFABP5 alone or combined with SB-FI-26, no mice with metastases were detected. These results represented significant (100%) suppression of metastasis by 100% when compared to the control group (Fisher's Exact test, *P* < 0.05) (Figure 3E and 3F). When tissues were stained histologically after autopsy, all mice in the control group developed metastases in the liver and lung, but not in bone. No histologically identifiable metastases were found in groups treated with dmrFABP5 or a combination of both inhibitors. Representative stained slides are shown in Figure 3G.

### Inhibitor dmrFABP5 suppressed tumorigenicity of PC3-M cells in nude mice in a similar way to PPARγ antagonist

**Figure 4:**
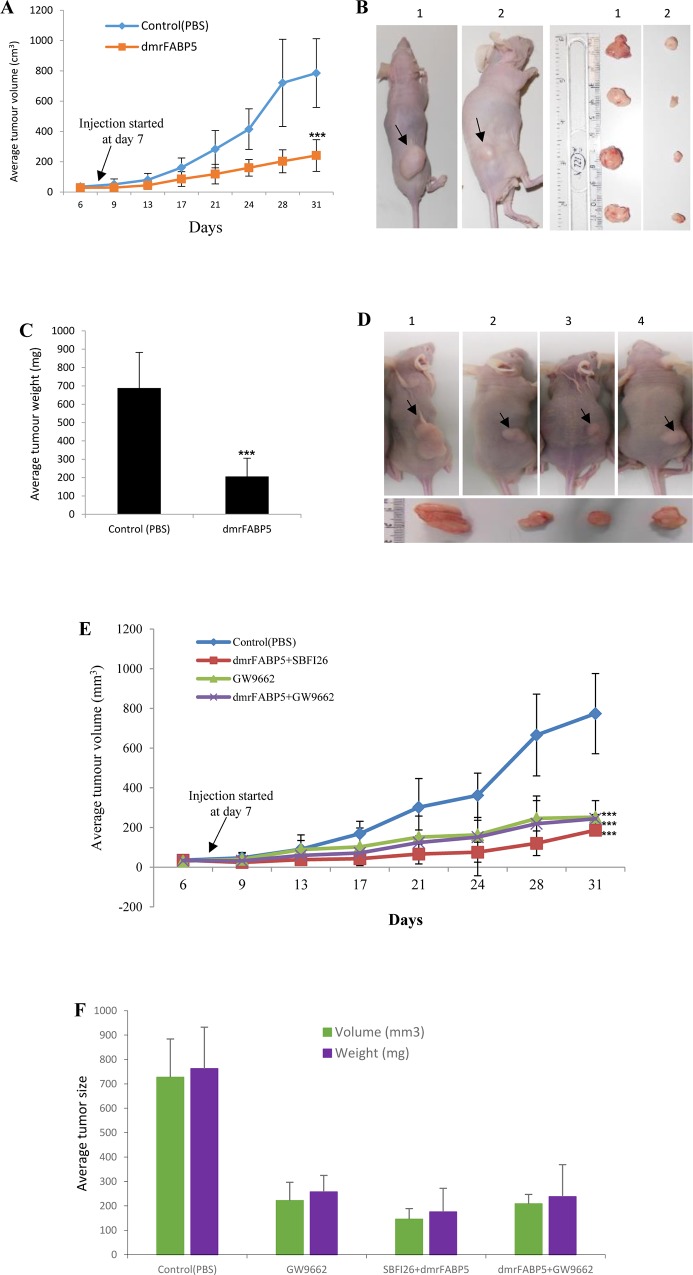
DmrFABP5 significantly suppressed tumorigenicity in prostate cancer xenografted mice **A.** Average volume of tumours produced by PC3-M cells in each group of mice treated with PBS (control) or dmrFABP5 (20mg/kg) for 31 days, starting on day 7 after inoculation (*n* = 8). **B.** Representative mouse and its corresponding tumours from control (1) or dmrFABP5 (2) groups. **C.** Average weight (mg) of tumours from control or treated groups of mice. **D.** Representative mouse and its corresponding tumours from each of the control (1), GW9662 (2), dmrFABP5 and SB-FI-26 (3) or the combination of dmrFABP5 and GW9662 (4) groups. **E.** Average weight (mg) of tumours in the control or experimental groups of mice. **F.** Average volumes (mm3) of tumours in the control or experimental groups of mice. In all tests, the difference between the control and each test group was assessed by 2-tailed unpaired Student's *t* test ^**^, *p* < 0.001; ^***^, *p* < 0.0001.

PC3-M cells were inoculated into the right flank of nude mice and the FABP5 inhibitor dmrFABP5 was injected subcutaneously to compare its tumor-suppressing effect with those of PPARγ antagonists and the chemical inhibitor SB-FI-26 (Figure 4). The average size of the tumours of the control group were larger than that of the test group by the third measurement on day 13 after the inoculation, and remained increasingly larger for the rest of the experiment (Figure 4A). On termination of the experiment or 31 days after inoculation of the cells in mice, the average tumor volume in the group treated with dmrFABP5 was 161± 61 mm^3^, compared to 627± 120 mm^3^ in the control group; a significant suppression of 75% (Student's t test, *p* < 0.001) (Figure 4A, and 4B). When tumors were weighed on termination at autopsy, the differences between control and treated groups were similar to those recorded by tumor volume (Figure 4C). To study the possible suppression by the PPARγ antagonist GW9662 and the possible synergistic effect of dmrFABP5 with either SB-FI-26 or GW9662, mice were treated from day 7 after inoculation in the following 3 ways: with dmrFABP5 plus SB-FI-26; with GW9662 or with dmrFABP5 plus GW9662 (Figure 4D). Similar to results observed in the first round of tumorigenicity studies, the average tumour size of the control group was larger than that of the test groups by the third measurement on day 13 after the inoculation, and remained increasingly larger until the end of the experiment (31days after the inoculation). Thus the average size of tumors (774 ± 202 mm^3^) in the control group was significantly larger than that in the 3 treated groups of mice (186 ± 25 mm^3^ in group treated with dmrFABP5 plus SB-FI-26; 252 ± 84 mm^3^ in the group treated with GW9662 alone; and 244 ± 22 mm^3^ in the group treated with dmrFABP5plus GW9662). These reductions by 76%, 67%, and 68%, were highly significant (Student's *t* test, *p* < 0.0001) (Figure 4E). When tumor weight was measured at autopsy, the differences between control and treated groups were similar to those measured by tumor volume (Figure 4F). There were no significant differences in the average tumor size of mice between any 2 of the 3 treated groups and the groups treated with dmrFABP5 alone (Student's t test, p>0.05).

### DmrFABP5 suppressed PPARγ activation

**Figure 5:**
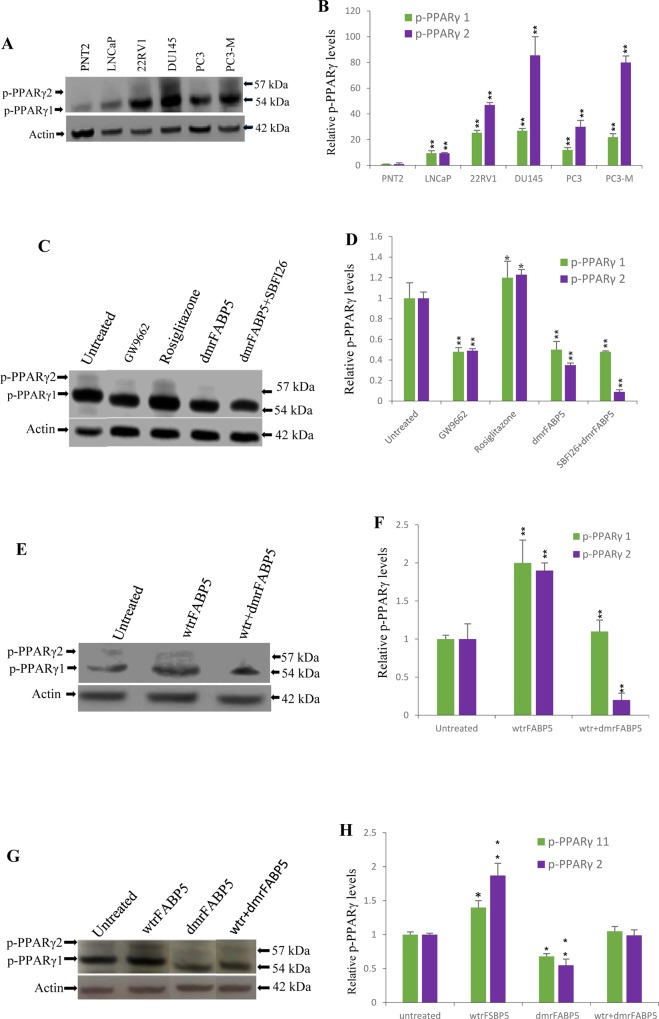
DmrFABP5 reduced the level of phosphorylated PPARγ (p-PPARγ1 and p-PPARγ2) in prostate cancer cells **A.** Western blot analysis of p-PPARγ1 and p-PPARγ2 in benign and malignant prostate epithelial cells. **B.** Quantitative assessment of the levels of p-PPARγ1 and p-PPARγ2 in benign and malignant prostate cancer cells. **C.** Effect of 24 h treatments with PPARγ antagonist (GW9662), PPARγ agonist (Rosiglitazone), dmrFABP5, or a combination of dmrFABP5 and SB-FI-26 on levels of p-PPARγ1 and p-PPARγ2 in PC3-M cells. **D.** Quantitative assessment: Levels of both p-PPARγ1 and p-PPARγ2 in untreated PC3-M cells were set at 1; levels in the other treated cells were obtained by comparison with those in untreated PC3-M. **E.** Effect of 24 h treatments with wtrFABP5 or with a combination of wtrFABP5 and dmrFABP5 on levels of p-PPARγ1 and p-PPARγ2 in LNCaP cells. **F.** Quantitative assessment: Levels of p-PPARγ1 and p-PPARγ2 in control 22RV1 cells were set at 1; levels in the other treated cells were obtained by comparison with those in control. **G.** Effect of 24 h treatments with wtrFABP5, or with dmrFABP5 on levels of p-PPARγ1 and p-PPARγ2 in 22RV1 cells. **H.** Quantitative assessment: Levels of both p-PPARγ1 and p-PPARγ2 in the control were set at 1; levels in the other treated cells were obtained by comparison with those in controls. For each Western blot, anti-β-actin was also used to normalize for possible loading errors. Results (mean ± SE) were obtained from three separate experiments and the differences were assessed by 2-tailed unpaired Student's *t* test. ^*^, *p* < 0.05; ^**^, *p* < 0.001.

The effect of dmrFABP5 on levels of phosphorylated PPARγ (p-PPARγ1 and p-PPARγ2, the presumed biologically active forms of PPARγ) was shown in Figure 5. Western blots using anti-p-PPARγ detected 2 bands representing the isoforms of p-PPARγ1 and p-PPARγ2 at 54 and 57kDa, respectively (Figure 5A). If the levels of p-PPARγ1 and p-PPARγ2 in PNT2 were both set at 1 and 1, relative levels in weakly malignant LNCaP, moderately malignant 22RV1, and highly malignant DU145, PC3 and PC3-M cells were 9.54 ± 1.81 and 9.5 ± 0.5; 25.4 ± 1.8 and 47.0 ± 1.7; 26.99 ± 1.72 and 85.5 ± 14.5; 12.08 ± 1.8 and 30 ± 5; 21.99 ± 2.63 and 80 ± 5, respectively (Figure 5B). Thus the levels of p-PPARγ, particularly p-PPARγ2, were significantly increased in all the malignant cell lines studied (Student's *t* test, *p* < 0.001). To investigate the effect of dmrFABP5 on p-PPARγ, PC3-M cells were treated for 24 hours with the PPARγ antagonist GW9662, with the PPARγ agonist Rosiglitazone plus dmrFABP5 and with the combination of dmrFABP5 and SB-FI-26 (Figure 5C). If levels of p-PPARγ1 and p-PPARγ2 in untreated cells were set at 1 and 1 (Figure 5D), the levels in cells treated with GW9662 and dmrFABP5 were reduced significantly by 52% and 51%; 50% and 65%, respectively (Student's *t* test, *p* < 0.001). Interestingly, cells treated with a combination of dmrFABP5 and SB-FI-26 dramatically reduced the level of p-PPARγ2 by 90%, and that of p-PPARγ1 by 52%. However, those mice treated with rosiglitazone significantly increased the levels of both p-PPARγ isoforms (Student's *t* test, *p* < 0.01) (Figure 5D). When LNCaP cells, which expressed very low levels of p-PPARγ1 and 2, were treated with wtrFABP5, the levels of both isoforms were increased by 1- and 0.9- fold, respectively. However, these increases were reversed completely by adding dmrFABP5 to the cells. Furthermore, addition of dmrFABP5 to LNCaP cells reduced the level of p-PPARγ2 by 5-fold (Student's *t* test, *p* < 0.001) to a level lower than that obtained in the pretreatment group (Figure 5E, 5F). When androgen-sensitive 22Rv1 cells were treated with wtrFABP5, with dmrFABP5 or with a combination of both (Figure 5G), wtrFABP5 significantly increased the levels of both p-PPARγ1 and 2 (Student's *t* test, *P* < 0.01) (Figure 5H). However, treatments with dmrFABP5 significantly suppressed the level of both PPARγ isoforms. Furthermore, the promoting effect of wtrFABP5 on levels of both p-PPARγ isoforms was completed blocked by treatment of mice with dmrFABP5.

### Fatty acid uptake by wtrFABP5 is not interrupted by dmrFABP5 in PC3-M cells

**Figure 6:**
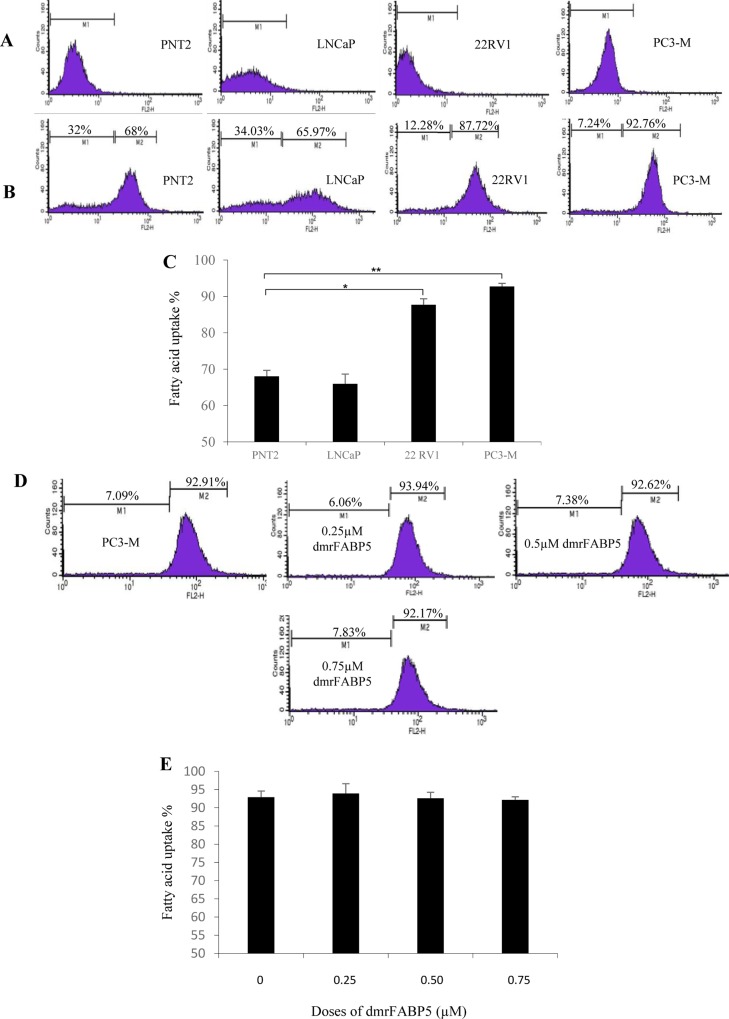
Fatty acid uptake by different prostate epithelial cell lines and the suppressive effect of dmrFABP5 in PC3-M cells **A.** Representative histograms for unstained PNT2, LNCaP, 22RVI and PC3-M cells without adding BODIPY-labelled fatty acid. The marker M1 highlights negative peaks of the subclass control. **B.** Representative histograms for fluorescence of stained PNT2, LNCaP, 22RVI and PC3-M cells 30 min after adding BODIPY-fluorescently labelled fatty acid; the marker M2 is placed to the right of M1 to highlight positive events (total percentage of cells with BODIPY-labelled fatty acid). **C.** Percentages of cells taking up BODIPY-labelled fatty acid from different prostate epithelial cell lines. **D.** Representative histograms for fatty acid uptake of PC3-M cells at a fixed concentration of BODIPY-labelled fatty acid in the presence of different concentrations of dmrFABP5. M1, unstained cells; M2, stained cells. **E.** Percentages of cells with fluorescently-labelled fatty acid in PC3-M control (untreated) and those wells treated with different concentrations of dmrFABP5 for 30 min with a fixed concentration of BODIPY-labelled fatty acid. Fluorescent intensity of each cell line was measured with an EPICS XL Cytometer (Beckman) at 570nm and data analysis was performed with SYSTEM II^™^ Software. Values were plotted as mean ± SE (error bars). The differences between the control and the experimental groups were assessed by 2-tailed unpaired Student's *t* test. ^*^, *p* < 0.01; ^**^, *p* < 0.001.

To investigate the possible effect of dmrFABP5 on fatty acid uptake of PC3-M cells, a fatty acid uptake assay was performed using red fluorescence-labelled fatty acid BODIPY and a cell analyser/sorter (Figure 6). Unstained cells (without BODIPY) were present in M1 zone (Figure 6A) and BODIPY stained cells were present in M2 zone after a 30min incubation (Figure 6B). In contrast to benign PNT2, significantly more than 20% and 25% of cells took up fatty acid in moderately malignant 22RV1 and highly malignant PC3-M (Student's *t* test *p* < 0.01 and *p* < 0.001) cells, respectively. Levels of uptake of fatty acids between benign PNT2 and weakly malignant LNCaP cells were similar (Figure 6C). The effect of increasing dmrFABP5 concentrations on fatty acid uptake in PC3-M cells was determined using a fixed concentration of BODIPY (Figure 6D). When PC3-M cells were incubated with dmrFABP5, cellular fatty acid uptake was not significantly (Students t-test, p>0.5) changed from 92.9% in the control, even when the concentration of dmrFABP5 was increased to 0.75 μM. Thus there was no reduction in fatty acid uptake in PC3-M cells produced by dmrFABP5 (Figure 6E).

### The level of p-PPARγ in dmrFABP5-treated tumours is greatly reduced

**Figure 7:**
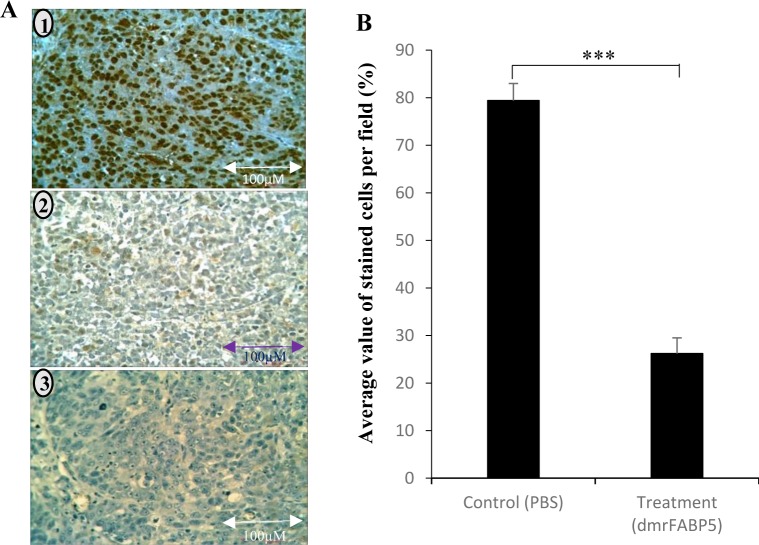
Immunocytochemical detection of p-PPARγ in tumours produced by PC3-M in nude mice PC3-M cells were orthotopically inoculated into prostate glands of the nude mice. The mice in the control group and those in the experimental group were injected peritoneally with PBS and dmrFABP5, respectively. **A.** Representative immunocytochemical staining of sections of the tumour tissues: 1) A tissue section from tumours of the control group treated with PBS. 2) A tissue section from tumours of the experimental group treated with dmrFABP5. 3) A tissue section from a tumour of the control group: the stain was blocked with a p-PPARγ blocking peptide as a negative control. **B.** Quantitative assessments of the average percentage numbers of the stained cells in control and in experimental groups. The difference between the control and the experimental group was assessed by 2-tailed paired Student's *t* test. ^***^, *p* < 0.0001.

The result of immunocytochemical staining is shown in Figure 7A. While strong nuclear staining was observed in tumour cells of the control group treated with PBS (1), very weak staining was seen in tumour cells of the experimental group treated with dmrFABP5 (2). The antibody specificity was confirmed when the stain was completely blocked the p-PPARγ-blocking peptides (3). When the staining intensity is expressed by the average percentage of the stained cells (Figure 7B); in control group, 79.4 ± 3.6% of the tumour cells were stained with the anti p-PPARγ antibody. Whereas in the experimental group, only 26.2 ± 3.3% of tumour cells were stained. Thus dmrFABP5 produced significant (Student t-test, *p* < 0.0001) reduction in the staining intensity of p-PPARγ in the tumour cells by 67%.

## DISCUSSION

Prostate cancer is the most common male cancer in the developed world and therefore it is one of the most serious healthcare issues. The main therapeutic strategy to treat prostate cancer patients for more than 4 decades has been ADT which targets AR and circulating male hormones [28]. However, once prostate cancer relapses with a more aggressive form to become CRPC, it no longer responds to ADT treatment effectively. There have been different hypotheses on how the androgen-dependent cells transform into androgen-independent cells. The main theory to date is that the biological sensitivity of AR is selectively amplified after the first round of ADT to such an extent that even micro-quantities of the remaining male hormone in peripheral blood can still promote the malignant progression of CRPC cells [29]. Thus treatment of patients with CRPC by further ADT is generally used in present clinical practice. However, contrary opinions to this practice have been expressed recently and it has been suggested that continued use of ADT in CRPC may lead to a therapeutic dead end [30, 31]. Our own study suggested that AR changes caused by ADT are not always relevant to malignant progression of CRPC cells. Thus targeting instead the FABP5- PPARγ-VEGF axis, which we suggest, gradually replaces the AR-mediated signalling pathway in tumour progression, could be an alternative way for treatment of CRPC [7]. In fact, a number of recent studies have confirmed the promoting role of PPARγ in malignant progression of prostate cancer and that the stimulation produced by FABP5- transported fatty acids plays an important role in PPARγ activation [32–34]. In this work, we have used the novel bio-inhibitor dmrFABP5 in nude mice to treat CRPC successfully by suppressing the biological activity of FABP5.

The mutant protein dmrFABP5 has a very similar structure to that of wtrFABP5, but the mutant is almost incapable of binding to fatty acids, as shown in Figure 1C and 1D. Here for the first time, we have shown that dmrFABP5 has a dominant negative effect on suppressing the biological activity of FABP5 and hence the tumorigenicity and metastatic ability of PC3-M cells. Thus, dmrFABP5 produced a significant suppression of PC3-M proliferation, invasiveness, migration and colony formation *in vitro*. When it was tested in nude mice, dmrFABP5 was highly effective in suppressing both the primary tumour in the prostate gland and the subcutaneous tumour in the flank. Thus dmrFABP5 produced on average a 13-fold reduction in tumour mass in the prostate gland and 3-fold reduction in mass of the subcutaneous tumours (Figures 3 and 4). Most importantly, dmrFABP5 produced a 100% suppression of metastasis in mice with CRPC cells implanted into the prostate gland (Figure 3). In our previous study, when the biological function of FABP5 was inhibited by SB-FI-26 in PC3-M cells, a significant decrease in cell proliferation, invasion, migration and anchorage-independent growth *in vitro* was observed [35–37]. SB-FI-26 showed efficient anti-tumour effects in both the mouse cell model for primary tumours implanted in the prostate gland (by 4.9- fold) and when inoculated in the flank (by 52%). Moreover, SB-FI-26 treatment of tumour-bearing nude mice suppressed metastases in 50% of these rodents. When dmrFABP5 was compared to SB-FI-26, the suppressive effect of dmrFABP5 was 2.7-fold higher in tumours growing in the prostate gland, and 30% higher in tumours growing in the flank. Furthermore, the suppressive effect of dmrFABP5 on metastasis in the mice was twice that of SB-FI-26. These results suggest that the suppressive effect of dmrFABP5 on tumour progression is much stronger than that of SB-FI-26. Thus this experimental therapeutic result for dmrFABP5 in our current study is a great improvement over that obtained by the chemically-synthesized inhibitor SB-FI-26 in our previous work [24].

In our previous report, the increased level of FABP5 plays an important role in promoting malignant progression in CRPC model cell systems by binding and transporting increased amounts of fatty acids which, in turn, stimulate the nuclear receptor PPARγ [16]. In this work we have shown that the treatment of PC3-M cells with the PPARγ antagonist, GW9662, produces a suppression of tumour growth similar to that obtained with dmrFABP5 (Figure 4). This result suggests that the suppressive mechanism of dmrFABP5 is related to the FABP5- PPARγ-signal transduction pathway [7]. Fatty acid-uptake results reported here show that the uptake of fatty acids is increased with increasing malignancy of the prostate cancer cells tested (Figure 6), indicating that enhanced amounts of fatty acids are taken up by the cancer cells and that at least part of them may be used to stimulate and thus activate PPARγ. In our previous work, SB-FI-26 produced a remarkable reduction in fatty acid uptake into PC3M cells. Thus it was suggested that SB-FI-26 may be a competitive inhibitor of fatty acids for FABP5 and hence prevent intra- and extra-cellular fatty acids from being transported into the cytoplasm [24]. The reduced uptake of fatty acids produced by SB-FI-26 may result in a reduction or cessation of the stimulation of PPARγ by fatty acids. Thus PPARγ may no longer be able to upregulate the down-stream cancer-promoting genes, such as VEGF, and to suppress those genes connected to apoptosis [7]. However, in our present work, dmrFABP5 does not produce any noticeable changes in uptake of fatty acids into PC3-M cells (Figure 6); a result which suggests that dmrFABP5 acts through a mechanism different from that of SB-FI-26.

Our recent study showed that it is the FABP5-PPARγ-VEGF signalling transduction axis, not the androgen receptor-initiated pathway, which is a dominant route for transduction of malignant signals in CRPC cells [7]. In this axis, the role of PPARγ is essential. Although we have found that the total PPARγ expressed in the malignant prostate cell lines is not higher than that in the benign PNT2 cells, both phosphorylated (presumed biologically-activated) PPARγ isoforms p-PPARγ1 and p-PPARγ2 [24] increase generally with increasing cellular malignancy. Both phosphorylated PPARγ isoforms are increased by wtrFABP5 and these increases can be inhibited by dmrFABP5 (Figure 5) in LNCaP cells which express low levels of phosphorylated PPARγ. Furthermore, the levels of the PPARγ isoforms in PC3-M cells are increased further by the PPARγ agonist, rosiglitazone or are greatly reduced by treatment with dmrFABP5, with GW9662, or with dmrFABP5 plus SB-FI-26 (Figure 5). These results suggest that dmrFABP5 may act to inhibit the stimulation of fatty acid transport induced by wild type FABP5 and thereby it can prevent activation or phosphorylation of PPARγ. This is supported by the immunocytochemical staining results (Figure 7) that dmrFABP5 caused significant reduction of p-PPARγ staining intensity in tumour tissues by 67%. PPARγ is a receptor for fatty acids localised in the nuclear membrane and this receptor plays an important role in promoting metastasis in prostate cancer [32]. Although both dmrFABP5 and SB-FI-26 suppress CRPC by affecting PPARγ, the inhibition of PPARγ phosphorylation by dmrFABP5 is unlikely to be caused by inhibition of fatty acid uptake in contrast to the inhibition observed in PC3-M cells treated with SB-FI-26.

It has been suggested that SB-FI-26 is a weak agonist of PPARγ in prostate cancer cells [21]. Since SB-FI-26 suppresses fatty acid uptake by displacing fatty acids which bind to FABP5, it is possible that some SB-FI-26 is delivered to activate PPARγ, but in a weaker way than with the fatty acids. This conclusion may be the reason why SB-FI-26 is a less effective inhibitor than dmrFABP5 in PC3-M cells. Since the inhibitory effects of dmrFABP5 and SB-FI-26 are exerted at 2 different points in the same signal transduction pathway, no further suppression is anticipated when they are used in combination. Since the exact route of fatty-acid transportation and delivery to PPARγ is not known, the reason why dmrFABP5 inhibits PPARγ is not fully understood. It has been suggested that actual physical interactions between FABPs and PPARs are necessary for an effective delivery of fatty acids [38]. Since the structure of dmrFABP5 is very similar to that of the wtFABP5, it is possible that dmrFABP5 occupies the same binding site as wtFABP5 in PPARγ and thereby prevents wtFABP5 from delivering fatty acids to PPARγ. Further study is needed to understand more fully the mechanisms involved in the suppressive effect of dmrFABP5 on PPARγ activation.

Previous work has suggested that the dependency of the prostate cancer cells on the FABP5-related pathway is increased gradually with a concomitant reduction in dependency on the AR-initiated pathway, until the former becomes completely dominant [7]. In this study, in androgen-responsive, moderately-malignant 22RV1 cells, dmrFABP5 produces a reduction in both phosphorylated isoforms of PPARγ by an average of 33.5% (Figure 5). This level of reduction is smaller than that of about 50% produced in the androgen-independent, highly malignant PC3-M cells. These results suggest that the proportion of phosphorylated PPARγ capable of regulation by the FABP5-related pathway is higher in PC3-M cells than that in 22RV1 cells. This result suggests that treatment by suppression of the FABP5-pathway is likely to be more effective in AR-negative CRPC cells. In conclusion, the novel FABP5 inhibitor dmrFABP5 suppresses the malignant progression of CRPC by blocking fatty acid-stimulation of PPARγ and thereby it curtails its up- or down-regulating effect on the down-stream cancer-promoting or -suppressing genes in the nude mouse model of CRPC.

## MATERIALS AND METHODS

### Construction of expression vectors

Construction of expression vectors was carried out as described previously [16]. After single and double mutations were generated by site-directed mutagenesis in the fatty acid-binding motif of human *FABP5* cDNAs, the wild type, the single- and the double-codon mutated *FABP5* cDNAs were first cloned into the pBluescript II SK (Qiagen) vector and then excised from the vector with restriction enzymes *Kpn*I and *Pst*I. The 3 cDNAs were then separately inserted into the bacterial expression vector pQE32 (Qiagen) which had been linearized with *Kpn*I and *Pst*I. The constructs were transformed into competent *E. coli* cells to form 3 separate bacterial pools which harboured cDNAs for wild type FABP5, single-point-mutated FABP5 and doubly- mutated FABP5. The correct orientations of the inserted cDNAs in the constructs were confirmed by nucleic acid sequence analysis. The recombinant proteins produced by these 3 pools of transformed *E.coli* cells were named as wild type recombinant FABP5 (wtrFABP5), singly-mutated recombinant FABP5 (smrFABP5) and doubly-mutated recombinant FABP5 (dmrFABP5), respectively. The final product smrFABP5 was obtained by changing Arg^109^ to Ala^109^ (R to A); and dmrFABP5 was obtained by changing Arg^109^ to Ala^109^ (R to A) combined with the changing of Arg^129^ to Ala^129^ (R to A) in the parental wtrFABP5.

### Expression and purification of recombinant FABP5s

The wild type and 2 mutant *FABP5* cDNAs were cloned into pQE32 expression vectors. The 3 recombinant FABP5s were produced in the BL21 strain of *E. coli* cells. After the initial incubation, the *E coli* cells were induced by 1mM IPTG. Bacterial samples were removed every hour for up to 6 hours after IPTG induction. Bacterial cells were harvested and lysed in lysis buffer, as described previously [16]. Recombinant FABP5s were separated from bacterial proteins by affinity chromatography on a Ni-NTA agarose column (Qiagen). Isolated proteins were dialyzed against PBS at 4˚C for 4 hours to remove imidazole and stored at −80˚C. The authenticity of the recombinant FABP5s was checked by Western blot analyses using both the Penta-His antibody (Qiagen) and the polyclonal rabbit anti-human FABP5 antibody (Hycult). Before the ligand binding assay, possible binding of fatty acids to wtrFABP5 was removed by delipidation using Lipidex-1000 (Sigma) [39].

### Ligand binding assay

The fatty acid-binding ability of all purified recombinant FABP5s was examined by the DAUDA displacement assay, which used fatty acids to displace the fluorescently-labelled fatty acid analogue DAUDA (Cayman). The excitation wavelength used for DAUDA was fixed at 345 nm, and the emission wavelengths for other molecules were collected over the range of 450-600 nm. Fluorescence-peak-emission wavelength for wtrFABP5 was determined by setting up the experiments as PBS, 3μM wtrFABP5, 2μM DAUDA and 3μM wtrFABP5 in 2μM DAUDA. Fatty acid-binding ability of the 3 FABP5s to 2μM DAUDA was tested by monitoring the change in fluorescent intensity and wavelength at peak emission [40] for each recombinant FABP5 in the present or absence of 2μM palmitic acid.

### Cell lines and chemical inhibitor SB-FI-26

The benign prostate cell line PNT2 [41], the highly-malignant, androgen-independent cell lines DU145[42], PC3 [43], and PC3-M [44], the moderately-malignant, androgen-responsive cell line 22RV1 [45], and the weakly malignant cell line LNCaP [46] were cultured and maintained in 1640 medium (Invitrogen) supplemented with 10% (v/v) FCS (Biosera), 100 U/mL penicillin and 100μg/mL streptomycin (Invitrogen). For LNCaP cells, 100μg/mL sodium pyruvate (Sigma) was added to the culture medium. The chemical inhibitor SB-FI-26 was purchased from ChemDiv, dissolved in DMSO, and stored at −20˚C. Before experimental work, a short tandem repeat (STR) profile was obtained on the genomic DNA from each cell line used. The authenticity of each cell line was confirmed when more than 80% of the STR profile matched that of DSMZ.

### Cell viability and proliferation assay

PC3-M cells (5×10^4^) were plated in triplicate in 96-well plates and incubated overnight at 37oC. Cells were treated with different concentrations of dmrFABP5 (5-70μM) and SB-FI-26 (25-125μM) for 24 h. Cell viability was assessed using the MTT assay, as described previously [16].

### Cell migration assay

The scratched wound healing migration assay was carried out to evaluate the effect of the inhibitor on the migration rate of PC3-M cells. Wounds were generated by scratching the cell monolayer with a blue pipette tip. The inhibitor dmrFABP5 was then added to the culture medium. The wound area was photographed under the microscope and resultant images assessed using ImageJ software.

### Invasion assay

This assay was performed as originally described [47] but with some modifications. PC3-M cells in serum-free medium were seeded in triplicate in the upper Boyden chamber (BD Biosciences) at a density of 2.5× 10^4^ cells per well. Complete medium was added to the lower chambers. After 3 hours of incubation at 370C, the inhibitors were added to the upper chambers. After 24 h of incubation, cells which invaded the lower chambers were stained with crystal violet and counted.

### Soft-agar assay

Low melting agarose was transferred to 6-well plates and after setting, 5×10^4^ cells/well were seeded on top of the set in 200μl of medium alone or media containing different FABP5 inhibitors. After 2 weeks incubation at 37°C, colonies larger than 300μm were counted in a similar way to that described previously [16].

### Nude mouse assay to test tumorigenicity and metastasis

PC3-M cells were transfected with the pGL4.50 [*luc2*/CMV/Hygro] vector (Promega) using FuGene HD transfection reagent (Promega) following the manufacturer's instructions. Individual colonies were isolated by ring cloning and 3 colonies that stably expressed the highest bioluminescent signals were identified using D-luciferin (Promega) with a Varioskan Flash Reader (Thermo Scientific). The relationship of the luminescent intensity with the number of cultured cells was assessed by an IVIS imaging system (Perkin Elmer). Cells (5×10^5^) from PC3-M- *luc2* colony were suspended in 30μL PBS and orthotopically implanted into 24, 8-10 weeks old male Balb/c nude mice (Charles River, UK) by direct injection into the dorsal prostate. Mice are too small in which to distinguish zonal structuresof the prostate [48]. One-week later, tumour-bearing mice were divided into 3 groups (8 each) and subjected to the following intraperitoneal injections: 1) control with PBS; 2) dmrFABP5 (20mg/kg); 3) dmrFABP5 (20mg/kg) plus SBFI26 (1mg/kg). Injections were repeated every two days for 25 days and the metastatic foci were monitored weekly using IVIS after mice were injected subcutaneously with D-luciferin (150 mg/kg). Bioluminescent images were analysed using the Living Imagine software (Xenogen) and recorded as total photons/second (p/s) within each defined region.

### Nude mouse tumorigenicity assay

PC3-M cells (2×10^6^) in 200μL PBS were injected subcutaneously into the right flank region of the mouse (6-8 week old) to compare the suppressive effects produced by dmrFABP5 and the PPARγ antagonist or agonist. In the first round, 2 groups of mice (8 each) were used: 1) control with PBS; 2) dmrFABP5 (20mg/kg); both groups were injected from day 7 after inoculation. In the second round, 4 groups of mice (5 each) were used and at day 7 after inoculation, each group was subjected to different intra-tumoural injections: 1) control with PBS; 2) PPARγ antagonist (GW9662, 1mg/kg); 3) dmrFABP5 plus SBFI26 (Sigma, UK); 4) dmrFABP5 plus GW9662. The injections were repeated every 2 days for 30 days, tumour size was measured every 3-4 days and the volume calculated using the formula of L×W×H×0.5236 [49]. All animal work was conducted in accordance with Cancer Research UK Guidelines under Home Office Project Licence PPL 40/3578 (to YK).

### Fatty acid uptake assay

Assay for uptake of fatty acids was performed using red fluorescently-labelled BODIPY [50]. The fluorescence intensity from cells before and 30 minutes after adding BODIPY was measured to determine fatty acid uptake. In inhibition and competition experiments, different concentrations of dmrFABP5 with the same concentration of labelled BODIPY were added directly to the highly-malignant PC3-M cells.

### Immunocytochemical staining for p-PPARγ

Histological section (cut at 4μm) slides were dewaxed and incubated with 1% BSA/PBS for ½ hr at room temperature to block background staining. The rabbit polyclonal anti p-PPARγ (#PA5-36763, Thermofisher) was applied at a dilution of 1:1500 in 1%BSA/PBS for 1hr at room temperature in a moisture chamber. Immunocytochemical staining was carried out using an enhanced HRP labelled polymer system, *EnVision+System, peroxidase (DAB)* (Dako Ltd, Ely, UK). The sections were then washed in running tap water before being counterstained in Mayers’ haemalum. They were then dehydrated through graded ethanol and xylene and were mounted in DPX (Merck, Poole, UK). For the blocked control, mix PPARγ blocking peptide (Santa Cruz #sc7273P) with p-PPARγ antibody at a final concentration of 0.2mg/ml with respect to peptide and 1:5000 with respect to antibody. Incubate at 37°C for 3hr followed by ON at 4°C. The staining intensity was calculated by the percentages of the stained cells. For each slide, four fields of 100-200 cells were counted.

### Statistical analysis

Student's *t*-test and Fisher's Exact test were carried out using GraphPad Prism software to compare the differences of the means between control and experimental groups. The difference is regarded as significant when *p* < 0.05; in the results, *p* value is represented by asterisks as follows: ^*^, *p* < 0.05; ^**^, *p* < 0.001; ^***^, *p* < 0.0001.

## Abbreviations

FABP: fatty acid-binding protein
PPARγ: Peroxisome proliferator-activated receptor gamma
CRPC: castration-resistant prostate cancer
AR: androgen receptor
ADT: androgen deprivation therapy
VEGF: vascular endothelial growth factor
